# The Relationship between Cancer Diagnosis Timing and Cognition in Older Adults in the National Health and Nutrition Examination Survey

**DOI:** 10.1002/alz.092394

**Published:** 2025-01-09

**Authors:** Annie Chen, Erin J Aiello Bowles, Jessica Chubak, Stephanie Cosentino, Jeanine M Genkinger, Deborah A Levine, Parisa Tehranifar, Katrin Wolfova, Sarah E. Tom

**Affiliations:** ^1^ Columbia University, New York, NY USA; ^2^ Kaiser Permanente Washington Health Research Institute, Seattle, WA USA; ^3^ University of Michigan, Ann Arbor, MI USA; ^4^ Charles University, Prague Czech Republic; ^5^ Columbia University Irving Medical Center, New York, NY USA

## Abstract

**Background:**

Cancer diagnosis is related to poor short‐term cognition, reflecting the condition, stress, and management. Less is known about long‐term relationships between time since cancer diagnosis and cognition. We evaluated the association between recency of cancer diagnosis and cognition.

**Method:**

The National Health and Nutrition Examination Survey is a nationally representative, cross‐sectional survey of community‐dwelling people in the United States. In 2011‐2012 and 2013‐2014, the study included questions on the timing of cancer diagnosis. Participants age ≥ 60 years completed the Digit Symbol Substitution Test and the Consortium to Establish a Registry for Alzheimer’s Disease Total Learning Test and Recall Test. Our analytic sample included individuals age ≥ 60 years who reported a cancer diagnosis and completed all cognitive tests. We calculated z‐scores for each cognitive test and a global score (sum across individual z‐scores). We used linear regression to assess the relationship between time since cancer diagnosis (current ‐5 years, >5 years) and cognition. We estimated an age and sex‐adjusted model and included potential confounders in a series of nested models, with each step adding: race and ethnicity, education level, marital status, and health insurance status. Analyses were weighted such that results represent the US community‐dwelling population.

**Result:**

The analysis sample included 477 participants who ever had a cancer diagnosis, excluding unknown and non‐melanoma skin cancers, corresponding to an implied population of 9,027,595 (including 11% melanoma skin, 23% breast, 16% prostate) with a mean age of 71.2 years (SD = 6.6). A total of 31% of participants reported a cancer diagnosis within the past five years. Demographic characteristics were similar based on cancer diagnosis timing. Adjusting for age and sex, participants greater than five years from cancer diagnosis had a higher cognition level, compared to those with a cancer diagnosis within five years (coefficient = 0.22, 95% CI = 0.05, 0.39). Results were similar when adjusting for additional covariates.

**Conclusion:**

Among older adults who ever had a cancer diagnosis, a diagnosis that occurred more than 5 years ago was related to better cognition. Future studies should investigate the roles of cancer site, treatments, and stage at diagnosis in this relationship.

